# Target of Rapamycin (TOR) Regulates the Expression of lncRNAs in Response to Abiotic Stresses in Cotton

**DOI:** 10.3389/fgene.2018.00690

**Published:** 2019-01-08

**Authors:** Yun Song, Linxuan Li, Zhaoen Yang, Ge Zhao, Xueyan Zhang, Lingling Wang, Lei Zheng, Fengping Zhuo, Huan Yin, Xiaoyang Ge, Chaojun Zhang, Zuoren Yang, Maozhi Ren, Fuguang Li

**Affiliations:** ^1^Zhengzhou Research Base, State Key Laboratory of Cotton Biology, Zhengzhou University, Zhengzhou, China; ^2^Institute of Cotton Research, Chinese Academy of Agricultural Sciences, Anyang, China; ^3^School of Life Sciences, Chongqing University, Chongqing, China

**Keywords:** target of rapamycin, expression pattern, stress response, cotton, long non-coding RNA

## Abstract

TOR (Target of Rapamycin) kinase is an evolutionarily conserved protein kinase, which integrates stress-related cues with growth and metabolic outputs. Long non-coding RNAs (lncRNAs) play a vital role in the regulation of eukaryotic genes. However, little is known about TOR's function in regulating the expression of lncRNAs in plants. In this study, four putative homologous genes encoding the TOR protein were identified by utilizing the recently completed cotton genome. Pharmacological experiments with TOR inhibitor AZD8055 and on silencing *GhTOR* genes resulted in obvious cotton growth retardation, indicating the conserved role of TOR in plant growth. The expression pattern analyses in different tissues reveal that TOR may play a role in root development, and the transcript levels of *TOR* genes were changed under different stress conditions. Importantly, we found TOR may be a key player in regulating the expression of long non-coding RNAs (lncRNAs). A total of 10,315 lncRNAs were discovered in cotton seedlings, 90.7% of which were long intergenic ncRNAs. Moreover, we identified the differentially expressed lncRNAs, of which 296 were significantly upregulated and 105 were downregulated in TOR inactivated plants. GO and KEGG analyses of differentially expressed lncRNA neighboring genes reveal that these differentially expressed lncRNA-targeted genes are involved in many life processes, including stress response, glutathione, and ribosomes in cotton. A series of differentially expressed lncRNAs potentially involved in plant stress response was identified under TOR inhibition. Collectively, these results suggest that cotton TOR proteins may directly modulate the expression of putative stress-related lncRNAs and eventually play a potential role in the cotton stress response.

## Introduction

TOR (Target of Rapamycin) is a highly evolutionarily conserved Ser/Thr protein kinase among eukaryotic species and modulates a broad spectrum of physiological and developmental processes (Rexin et al., 2015; Dobrenel et al., 2016). TOR protein possesses five highly conserved domains represented by HEAT repeats, the FAT domain, the FRB domain, the Kinase domain and the FATC domain from N- to C-terminus (Xiong and Sheen, 2015). In yeast and animals, TOR acts in two functionally distinct complexes: the rapamycin-sensitive TORC1 complex and rapamycin-insensitive TORC2 complex (Loewith et al., 2002; Wullschleger et al., 2006). However, no equivalents of the TORC2 specific proteins, for instance RICTOR and SIN1, are present in the plant genome databases (Rexin et al., 2015). Rapamycin (RAP), known as the most specific TOR inhibitor, has been widely used to dissect TOR functions in yeast and mammals. Nevertheless, most of the examined plants are RAP-insensitive (Xiong and Sheen, 2015). Thus, a new generation of ATP-competitive chemical inhibitors specific to TOR kinase such as Torin1, Torin2, KU0063794, and AZD8055 (AZD) have been applied for TOR studies in yeasts, plants, animals, and humans (Feldman et al., 2009; García-Martínez et al., 2009; Chresta et al., 2010; Liu et al., 2011; Xiong et al., 2017). Among these inhibitors, AZD was the strongest inhibitor and has been widely used in plant TOR research (Dong et al., 2015; Li et al., 2015b; Deng et al., 2016; Song et al., 2017; Xiong et al., 2017).

As the prime resource of textile fiber and an important oilseed crop, cotton is widely cultivated around the world (Paterson et al., 2012; Wang et al., 2012). In our previous study, we have investigated the TOR signaling pathway in tetraploid cotton (Song et al., 2017). The TOR signaling pathway is also present in cotton and plays a vital role in cotton growth and development. The major signaling components TORC1-S6K-RPS6 are conserved and further expanded in the cotton genome (Song et al., 2017). The core components of the TORC2 complex are absent in the cotton genome (Song et al., 2017). Cotton seedlings are insensitive to rapamycin, which is similar to the observations in *Arabidopsis* (Ren et al., 2012; Xiong et al., 2017). However, the second-generation TOR inhibitor AZD can significantly suppress cotton growth in a dose-dependent manner (Song et al., 2017).

In previous studies, TOR has also been shown to be a master regulator of plant stress response. For example, in *Arabidopsis*, TOR interacting with RAPTOR1, a conserved component of TORC1, regulates the activity of S6K1 and responds to osmotic stress (Mahfouz et al., 2006). Researchers also found that *AtTOR* expression could relieve the inhibition of primary root growth exposed by excess nitrogen (Deprost et al., 2007). Ectopic expression of *AtTOR* in rice improves water-use efficiency, further indicating the significant influence of TOR on plant responses to abiotic stress (Bakshi et al., 2017). Phytohormone abscisic acid (ABA) plays a critical role in integrating a wide range of stress signals and controlling downstream stress responses. TOR kinase can directly phosphorylate ABA receptor PYL to prevent activation of the stress response, and their reciprocal regulation eventually balances plant growth and stress response (Wang et al., 2018a). Except for response to abiotic stress, the roles of TOR in plant defense against microbial pathogens were investigated recently (De Vleesschauwer et al., 2018).

In the high-throughput sequencing data, some unexpected transcribed genomic regions which show no or weak protein coding capacities were regarded as “dark matters” (Derrien et al., 2012; Iaconetti et al., 2013; Ma et al., 2014). In the last decades, researchers have studied these “dark matters” and found that they had some specific functions. As one part of the “dark matters,” long non-coding RNAs (lncRNAs) were reported to be vital components of eukaryotic gene regulation, genome stability and nuclear domain organization (Mercer et al., 2009; Wang and Chang, 2011; Chekanova, 2015). It is generally defined that lncRNAs are transcripts longer than 200 bp in length. On the basis of their genomic origins, lncRNAs can be broadly classified into three types: long intergenic ncRNAs (lincRNAs), intronic ncRNAs (incRNAs) and natural antisense transcripts (NATs) transcribed from the complementary DNA strand of the associated genes (Chekanova, 2015; Mattick and Rinn, 2015). In *Arabidopsis*, almost 40,000 putative lncRNAs including over 30,000 NATs and over 6,000 lincRNAs were identified in the previous studies (Liu et al., 2012; Jin et al., 2013; Wang et al., 2014a). The majority of lncRNAs are transcribed by RNA Pol II, and some lncRNAs can also be produced by two additional plant-specific RNA polymerases, Pol IV and Pol V (Wierzbicki et al., 2008; Li et al., 2014a). The molecular functions of lncRNAs have been widely investigated in recent research. They can regulate gene expression through various mechanisms (Chekanova, 2015). LncRNAs can mimic the targets of regulatory proteins, thus preventing regulatory proteins from binding to DNA or RNA (Franco-Zorrilla et al., 2007). They can also regulate the Pol II transcription machinery directly (Wang et al., 2014b). Researchers also found that the long non-coding RNAs functioned as endogenous target mimics for microRNAs in plants including rice and *Arabidopsis* (Wu et al., 2013).

In recent decades, many studies have uncovered quite a number of lncRNAs and their functions in plants. In *Arabidopsis*, genome-wide analysis uncovered 6,480 long intergenic non-coding RNAs (Liu et al., 2012). In maize, Li et al., identified 20,163 putative lncRNAs and more than 90% were predicted to be the precursors of small RNAs (Li et al., 2014a). In cotton, the lncRNAs were reported to be involved in fiber development and drought stress responses (Wang et al., 2015a; Lu et al., 2016; Zou et al., 2016). Through global analysis of ribosome-associated non-coding RNAs in the *Arabidopsis* roots, scientists unveiled new modes of translational regulation (Bazin et al., 2017). Long-non-coding RNA PMS1T produced phased small-interfering RNAs and regulated photoperiod-sensitive male sterility in rice (Fan et al., 2016). The *Arabidopsis* non-coding RNA HID1 acted through phytochrome-interacting factor 3 (PIF3) and mediated control of photomorphogenesis by red light (Wang et al., 2014). The *Arabidopsis* long intergenic non-coding RNA (lincRNA) APOLO participates in the spatial association and interaction between APOLO and the distant PID genomic regions via formation of a dynamic chromatin loop that determines PID expression (Ariel et al., 2014). Scientists identified an antisense long non-coding RNA TWISTED LEAF (TL) and found that TF regulated R2R3-MYB gene expression and maintained leaf blade flattening (Liu et al., 2018). In rice, researchers revealed that overexpressing lncRNA LAIR could increase the grain yield and regulate the expression of neighboring gene cluster expression (Wang et al., 2018b). Tomato fruit ripening was altered by CRISPR/Cas9-mediated mutagenesis of lncRNA1459 (Li et al., 2018).

Until now, the relationship between TOR kinase and lncRNAs was only elucidated in animals. The transcript levels of lncRNA growth-arrest specific 5 (GAS5) can be enhanced by mTOR inhibition in certain prostate cancer cell lines (Yacqub-Usman et al., 2015). A novel mTOR activator can greatly decrease the level of a long non-coding RNA (lncRNA) known as FLJ11812, which binds with MIR4459 and targets ATG13 (Ge et al., 2014). The lncRNA Colorectal Neoplasia Differentially Expressed (CRNDE) is mainly expressed in human brains, and overexpression of specific CRNDE transcripts can promote cell growth. Studies revealed that the expression level of CRNDE could be modulated by mTOR signaling (Wang et al., 2015b). Researchers also found that lncRNA Urothelial Carcinoma-Associated 1 (UCA1) played a positive role in cancer cell glucose metabolism, which was performed through the cascade of mTOR-STAT3/microRNA143-HK2 (Li et al., 2014b). UCA1 can also enhance tamoxifen resistance in breast cancer cells via inhibiting the mTOR signaling pathway (Wu and Luo, 2016). Compared with extensive research about the protein coding gene affected by TOR (Ren et al., 2012; Dong et al., 2015; Deng et al., 2016; Xiong et al., 2016; Song et al., 2017), it remains largely unexplored whether and how the TOR kinase protein regulates lncRNAs, especially in plants.

In the present study, we identified *TOR* genes in *Gossypium hirsutum* and characterized the functions of TOR proteins in cotton by AZD pharmacological experiments and silencing of *GhTOR* genes by VIGS (virus-induced gene silencing) technology. The expression pattern analysis under different tissues and stresses suggested that TOR may play a role in cotton stress response. Genome-wide analysis of lncRNAs in the TOR-inactivated plants revealed that the stress-related neighboring genes of differentially expressed lncRNAs were significantly enriched. We further explored the functions of lncRNA candidates by differential expression analysis under stresses in cotton. As an economically important fiber and oil crop cultivated in many tropical and subtropical areas of the world, cotton is constantly exposed to a range of abiotic and biotic stresses, including drought, extreme temperature, high salinity and fungal pathogen infections (Mahajan and Tuteja, 2005; Qanmber et al., 2018). Cotton production is significantly influenced by these stresses. Therefore, it is necessary to uncover the underlying mechanism of plant stress response. Collectively, our observations showed the close links between TOR signaling and lncRNA expression in cotton. These results provide new insights into the functions of TOR signaling in plants.

## Materials and Methods

### Sequence Identification and Gene Structure Analysis of *TOR* Genes in *Gossypium hirsutum*

The *Gossypium hirsutum* genome sequences and the proteome sequences were downloaded from the CottonGen database (http://www.cottongen.org) (Yu et al., 2014). The amino acid sequences of TOR from *A. thaliana*, which were acquired from The *Arabidopsis* Information Resource version 10 (TAIR 10) (http://www.arabidopsis.org), were used as query sequences to identify complete TOR members in the *G. hirsutum* protein database through the blastp program. The full-length protein sequences of *Arabidopsis* and *G. hirsutum* TOR were aligned with Clustal W, and MEGA 5.0 software was applied to construct a neighbor-joining phylogenetic tree using the bootstrap method with 1,000 replicates (Tamura et al., 2011). The positions of the exons and introns were acquired from the gff3 file using a Perl script, and the online tool GSDS 2.0 was used to display the gene structure (Hu et al., 2015).

### Plant Materials and Growth Conditions

In this study, upland cotton (*Gossypium hirsutum* L. cv CCRI24) was used. The cotton seeds were surface-sterilized according to the method described previously (Yang et al., 2014). The sterilized seeds were germinated on a Murashige and Skoog (MS) medium (with 1% agar) supplemented with DMSO (as a control) or 5 μM AZD (Selleckchem, Houston, TX, USA). Then they were grown in a controlled environment at 25°C under 16 h continuous light and 8 h darkness for 4, 6, and 8 days, respectively. These cotton seedlings were photographed. The primary root lengths, fresh weights and lateral root numbers were measured manually. Three biological experiments were performed, and each consisted of 20 plants per treatment. The data was expressed as the mean ± SD of three independent experiments.

### VIGS Experiments

The conserved cDNA fragments of *GhTOR* genes were amplified and cloned into the pTRV2 vectors according to the method described in the previous study (Liu et al., 2002). The recombinant plasmids were transformed into *A. tumefaciens* strain GV3101. Cotton VIGS experiments were performed following the previously described procedure (Pang et al., 2013). Cotyledons of 7-day-old CCRI24 cotton seedlings were used for the injections. All plants infiltrated with the empty pTRV2 were used as the negative controls in this research. Plants infiltrated with the pTRV2 vector containing the cDNA fragment of *GhPDS* gene were used as the positive controls to test the efficiency of VIGS, and the albino phenotype was examined (Pang et al., 2013). Three biological experiments were performed, and each consisted of 10 plants per treatment. The data was expressed as the mean ± SD of the three independent experiments.

### Gene Expression Analysis and Heatmap

The publicly available transcriptome data of TM-1 was used to assess the relative gene expression patterns (Zhang et al., 2015). RNA-Seq data were acquired and analyzed as described in the previous report (Yang et al., 2017; Qanmber et al., 2018). A heatmap was drawn by the MeV (Multiple Experiment Viewer) software (Saeed et al., 2003).

### LncRNA Identification

Four-day-old cotton seedlings were transferred into the MS medium containing DMSO and 5 μM AZD. The seedlings were collected at 24 hours post-treatment, frozen immediately in liquid nitrogen and stored at −80°C. Total RNA was extracted from samples using the RNAprep pure kit (Tiangen, Beijing) for RNA sequencing. Sequencing library preparation followed our previously described procedure (Song et al., 2017). Three biological replicates were sequenced using the Illumina Hiseq 2500 for each treatment. All raw sequencing data used in the current study have been deposited in the Sequence Read Archive database under accession number SRX2568809. The lncRNAs were identified from the novel and antisense transcripts of transcriptome assemblies by adopting the following steps: (1) the transcripts with low expression levels and with only a single exon were removed while the transcripts with two or more exons were selected; (2) the transcripts with length < 200 bp were removed; (3) the transcripts were searched against the Cuffcompare database to eliminate transcripts encoding proteins and protein coding domains; (4) the transcripts with FPKM (expected number of Fragments Per Kilobase of transcript sequence per Millions base pairs sequenced) < 0.5 were removed; (5) the transcripts that failed to pass any one of the protein-coding-score tests using the Coding Potential Calculator (CPC), Coding-Non-Coding Index (CNCI) and Pfam Scan software were regarded as TUCP (transcripts of uncertain coding potential) (Kong et al., 2007; Sun et al., 2013; Finn et al., 2014). The optimized transcripts with no protein-coding potential were identified as lncRNAs and were listed in Supplementary Table 1.

### Localization of lncRNAs and Protein-Coding Genes in Cotton Genome

The information about protein-coding genes and chromosomes was obtained according to the CottonGen database for cotton research (Yu et al., 2014). The program Circos was used to generate a diagram that showed the localization, abundance and expression level of lncRNAs and protein-coding genes in the cotton genome (Krzywinski et al., 2009).

### Differential Expression of lncRNAs Between DMSO and AZD Treated Cotton Seedlings

The Cuffdiff software was used to identify the differentially expressed lncRNAs between DMSO control and AZD treatment (Trapnell et al., 2012). The fold changes were calculated via Log_2_ (FPKM_AZD_/FPKM_DMSO_). LncRNAs with a |Log_2_(Fold change)|≥1 and adjusted *P* ≤ 0.05 were selected as differentially expressed lncRNAs and displayed in Supplementary Table 2. The co-location protein-coding genes, which were upstream or downstream 100 kb away from lncRNAs, were used to analyze the GO (Gene Ontology) and KEGG (Kyoto Encyclopedia of Genes and Genomes) terms. The Pearson correlation coefficient was employed to explore the expression relationship between these lncRNAs and the neighboring protein-coding genes, and the lncRNAs with r_p_ > 0.95 were selected. GO analyses of the differentially expressed lncRNAs were performed by the GOseq program (Young et al., 2010). KOBAS software was used to test the enrichment of differentially expressed lncRNAs in KEGG pathways (Mao et al., 2005; Kanehisa et al., 2008).

### Quantitative Real Time PCR (QRT–PCR)

Total RNA isolation and the generation of first-strand cDNA were performed as previously described (Song et al., 2017). The CFX96 system (BIO-RAD) was used to perform the QRT-PCR experiments by using an SYBR Premix Ex Taq II (TAKARA) kit. The primers used for QRT-PCR were designed by Primer Premier 5.0 and presented in Supplementary Table 3. Three biological replicates were performed and the reactions were performed in triplicate for each run. Expression levels of the target lncRNAs were normalized to *GhHISTONE3* in cotton. The 2^−ΔΔ*Ct*^ analysis method was used to measure the relative expression levels of lncRNAs (Livak and Schmittgen, 2001). ANOVA analysis and a Tukey-Kramer multiple comparison test were used for data analysis. *P* < 0.05 was considered as statistically significant.

## Results

### Identification, Structure, and Domain Analysis of *Gh*TOR

**Figure 1:**
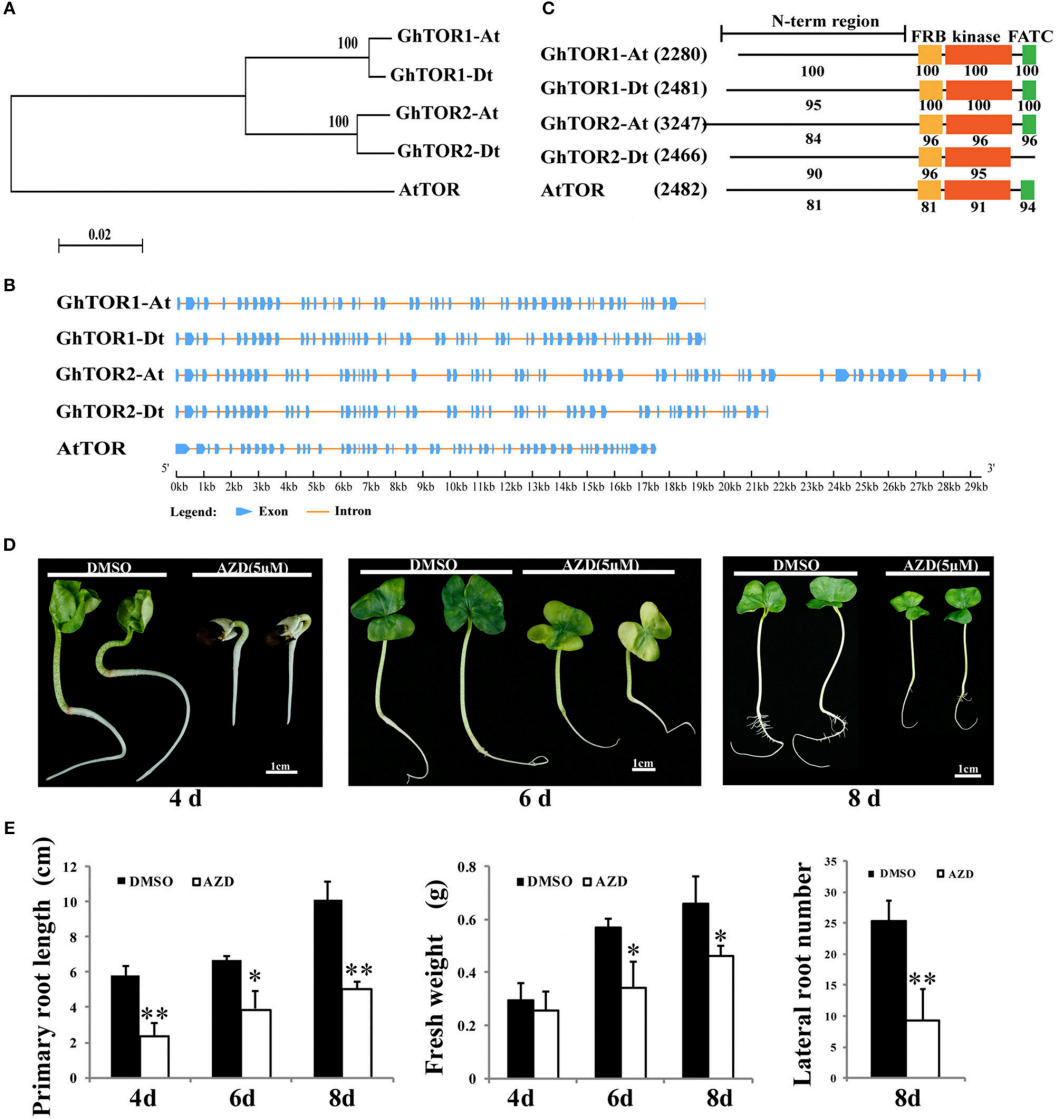
Phylogenetic analysis of *Gh*TOR and pharmacological experiments. **(A)** Phylogenetic analysis of GhTORs and AtTOR using a neighbor-joining method. **(B)** Gene structure analysis of *GhTOR* genes. The number, position, and length of exons and introns within *GhTOR* genes are displayed. Boxes and lines indicate the exons and the introns, respectively. **(C)** Comparison of the conserved domains between GhTOR and AtTOR proteins. The number in brackets represents the amino acid number of every protein. Each value indicates the percentage of identity with the corresponding domain sequences of GhTOR1-At. **(D)** TOR inhibition by AZD significantly retarded cotton seedling growth. Sterile cotton seeds were germinated on plates with DMSO and AZD (5 μM) for different days. Cotton seedlings grown in MS medium containing DMSO and AZD (5 μM) for 4, 6, and 8 days were photographed, respectively. Bars = 1 cm. **(E)** The primary root lengths, fresh weights and lateral root numbers of **(D)** were measured. Each experiment contains three biological replicates. Error bars indicate ±SD for triplicates. Significant differences from the control are indicated by ^*^P < 0.05, ^**^P < 0.01.

The TOR protein sequences of *Arabidopsis* were used as queries to search *G. hirsutum* database (Yu et al., 2014) using the blastp program with a cut-off E-value of 1e-5. Thereafter, four candidates were identified, and their sequences were extracted (Table 1). On account of the gene number and chromosome locus, the four *GhTOR* genes were designated as *GhTOR1-At, GhTOR1-Dt, GhTOR2-At*, and *GhTOR2-Dt*. Compared with the single *TOR* gene in diploid *Arabidopsis*, the number of *TOR* genes in allotetraploid cotton was significantly expanded. To dissect the evolutionary relationship of *TOR* genes between *G. hirsutum* and *Arabidopsis*, a phylogenetic tree was constructed by MEGA 5.0 software using the NJ method (Figure 1A). As shown in Figure 1A, *GhTOR* genes can be divided into two groups, and every group has two orthologs according to the phylogenetic tree, indicating that cotton *TOR* genes may have undergone duplication because only one *AtTOR* was found. Gene function is closely related with its structure; thus, we analyzed the gene structure of *GhTOR*s (Figure 1B). Multiple-exons (50 to 66 exons) were displayed in *GhTOR* genes. We also found that *GhTOR1-At* and *GhTOR1-Dt* had highly similar exon patterns, and this was also the case between *GhTOR2-At* and *GhTOR2-Dt*. Similar domain organizations were also found between GhTORs and AtTOR proteins, except for GhTOR2-Dt, which lacked the FATC domain (Figure 1C). Collectively, these observations indicated that GhTORs have a close phylogenetic relationship with AtTOR and confirmed that TOR is also conserved in *G. hirsutum*.

**Table 1:** Gene loci information pertaining to cotton *TOR* genes.

**Gene name**	**Gene locus**	**Chr**	**Direction**	**Start**	**End**
GhTOR1_At	Gh_A01G0097	A01	-	831404	850685
GhTOR1_Dt	Gh_D01G0092	D01	-	735395	754726
GhTOR2_At	Gh_A12G0159	A12	+	2336388	2365784
GhTOR2_Dt	Gh_D12G2836	D12	-	52166	73797

### Suppression of TOR by AZD Significantly Inhibited the Growth of Cotton Seedling

AZD is a novel ATP-competitive specific inhibitor of the catalytic site of TOR kinase and has been successfully applied to research in the higher plants by independent groups (Montané and Menand, 2013; Dong et al., 2015; Li et al., 2015b; Xiong et al., 2017). AZD can directly bind to the kinase domain of TOR protein to inhibit the activity of both TORC1 and TORC2 (Chresta et al., 2010; Benjamin et al., 2011). AZD has shown relatively higher potency than other TOR inhibitors in both animals and plants (Montané and Menand, 2013; Dong et al., 2015). Thus, AZD was selected to dissect the biological functions of TOR in cotton (Figure 1D). Cotton seeds were germinated on the MS medium containing DMSO (as a control) or 5 μM AZD for 4, 6, and 8 days, respectively. These TOR-suppressed cotton seedlings showed a significant delay in the transition from heterotrophic to photoautotrophic growth compared with the cotton seedlings exposed to DMSO. The fresh weights and root lengths of TOR-suppressed cotton seedlings at 4, 6, and 8 days were only 86%, 60% and 69% of control seedlings, respectively (Figure 1E). Moreover, the number of lateral roots in the TOR-inhibited cotton seedlings also showed a significant decrease compared to that of the control seedlings. These data indicated that TOR plays a vital role in cotton growth.

### Knockdown of *GhTOR* Expression Causes Retarded Growth in Cotton

**Figure 2:**
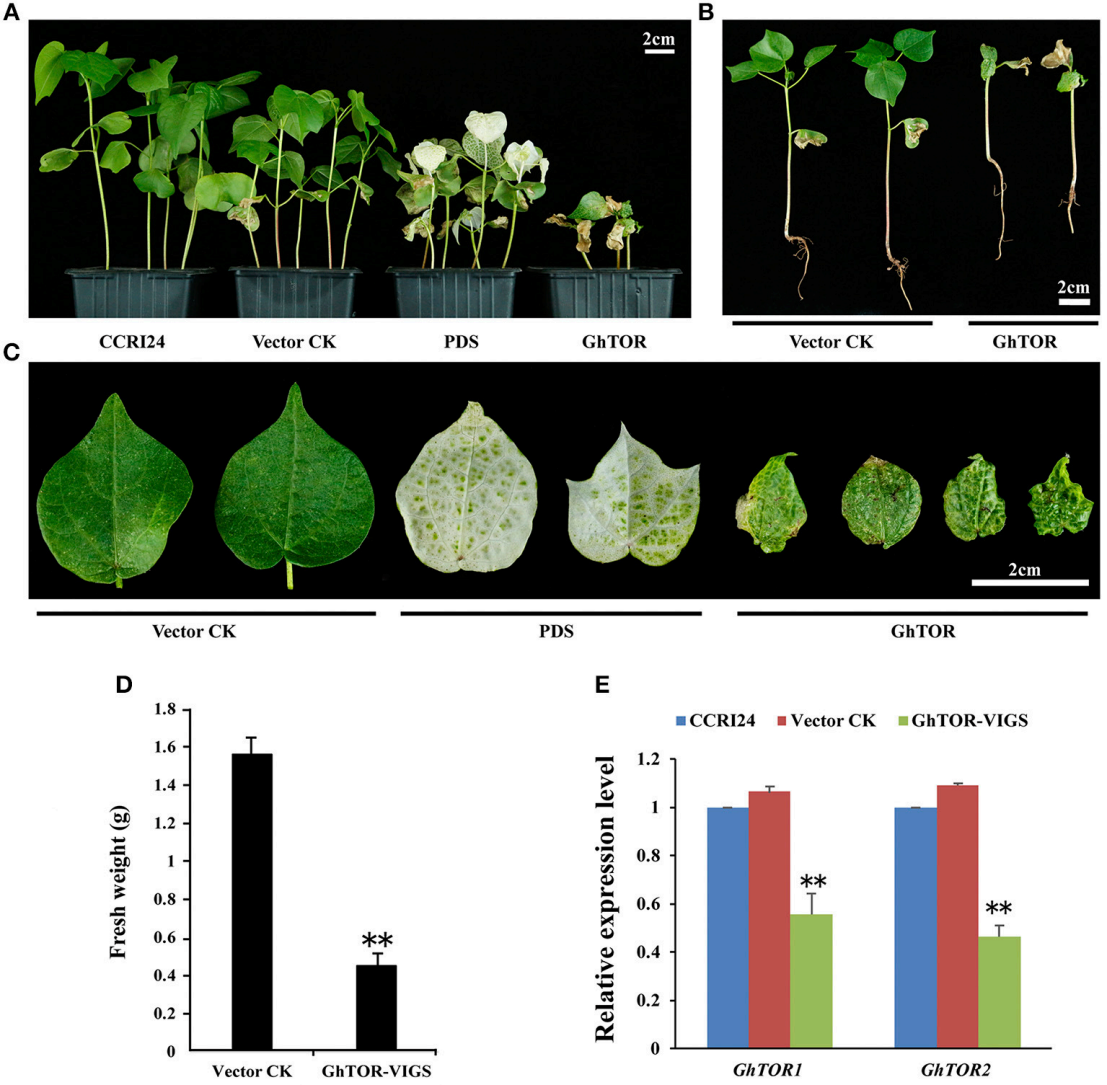
Phenotypes of *GhTOR* VIGS cotton plants. **(A)** Potting of wild type plants (CCRI24), and plants infiltrated with vector control (*TRV:00*), vector containing *GhPDS* (*TRV:GhPDS*) and vector containing *GhTOR* (*TRV:GhTOR*). Bar = 2 cm. **(B)** The whole vector control and *GhTOR* silenced cotton seedlings. Bar = 2 cm. **(C)** Close-up of vector control, *GhPDS*-silenced and *GhTOR*-silenced cotton leaves. Bar = 2 cm. **(D)** Fresh weight of the control and *GhTOR*-silenced cotton plants. Each experiment contains three biological replicates, and every replication contains at least twenty plants. Error bars indicate ±SD for triplicates. Significant differences from the control are indicated by ^**^P < 0.01. **(E)** Decreased *GhTOR* expression levels in VIGS plants. Total RNA was extracted from leaves at 14 days post-agroinfiltration. The expression level of *GhTOR1* (*GhTOR1-At* and *GhTOR1-Dt*) and *GhTOR2* (*GhTOR2-At* and *GhTOR2-Dt*) in VIGS plants was compared with that in wild type plants (CCRI24) and plants infiltrated with vector control. Asterisks denote significant difference compared with wild-type plants (CCRI24) (^**^P < 0.01).

To further investigate the cellular function of cotton TOR signaling, virus-induced gene silencing (VIGS) was used in this study (Liu et al., 2002; Pang et al., 2013). The conserved cDNA fragment of the four *GhTOR* genes was cloned into the virus vector pTRVRNA2 (pTRV2). The empty vector pTRV2 and vector pTRV2 containing the cDNA of *GhPDS* were used as negative and positive controls, respectively. These vectors were transformed into *Agrobacterium* cells and then inoculated into the cotyledons of the wild-type cotton CCRI24. As shown in Figures 2A–C, most of the growth of *GhTOR*-silenced plants was significantly retarded compared with that of the control seedlings. We found that most of the *GhTOR*-silenced plants exhibited pale yellow-green and shrinking leaves under normal growth conditions (Figure 2C), and the phenotype was consistent with the phenotype displayed in AZD-treated cotton seedlings (Figure 1D). A small part of *GhTOR*-silenced plants' leaves showed completely wilting phenotypes, which could be due to the lethal phenotype caused by the complete silence of *GhTOR*. The fresh weights of TOR-silenced cotton were also significantly reduced (Figure 2D). To validate that these phenotypes were caused by the reduced expression of the *GhTOR* genes, the expression levels of *GhTOR* genes were analyzed by QRT-PCR (Figure 2E). The top leaves of the cotton seedlings indicated in Figure 2A were harvested to perform QRT-PCR, respectively. Primers that targeted the conserved sequences of *GhTOR1* (*GhTOR1-At* and *GhTOR1-Dt*) and *GhTOR2* (*GhTOR2-At* and *GhTOR2-Dt*) were designed. The expression levels of *GhTOR1* and *GhTOR2* genes both decreased consistently in *GhTOR*-silenced plants. Our results confirmed that the retarded growth phenotype was caused by the reduced expression of the *GhTOR* genes.

### Analysis of *GhTOR* Gene Expression Pattern in Different Tissues

Gene expression pattern is helpful to dissect gene biological function. To realize the biological function of TOR in cotton, we detected the expression patterns of the *TOR* genes using the publicly available transcriptome data of *Gossypium hirsutum* (Supplementary Figures 1A,B) (Li et al., 2015a). As shown in Supplementary Figure 1, the expression profiles of the orthologs between the *GhTOR1-At, GhTOR1-Dt*, and *GhTOR2-Dt* were similar in different tissues during seed germination, seed (fiber) development and root development, suggesting that their biological functions might be conserved. We found that all the *GhTOR* genes were widely expressed in the vegetative (root, stem and leaf) and reproductive (torus, petal, stamen, pistil, calycle and −3, −1, 0, 1, 3, 5, 10, 20, 25, and 35 days post-anthesis (DPA) ovule) tissues as well as in the fiber (5, 10, 20, and 25 DPA), suggesting that *TOR* genes have multiple biological functions in different tissues (Supplementary Figure 1A). *GhTOR2_At/Dt* have relatively low expression levels in cotton petals and stamens. *GhTOR1_At/Dt* were highly expressed in the stem, leaf and the late stage of ovule development (20 DPA and 25 DPA fibers). *GhTOR1_At/Dt* were highly expressed in seeds and roots at different sampling stages (Supplementary Figure 1B), indicating that *GhTOR1_At/Dt* play a potential role in the seed germination and root development.

### Genome-Wide Identification and Characterization of lncRNAs in *Gossypium hirsutum*

LncRNAs can regulate gene expression through various mechanisms (Chekanova, 2015). The pair-end RNA-seq database has become a major resource to discover lncRNAs (Ilott and Ponting, 2013). To further understand TOR functions, paired-end RNA-Seq data of transcripts from CCRI24 and AZD-treated CCRI24 were used to find novel lncRNAs in *Gossypium hirsutum* seedlings. After data quality control, TOPHAT2 mapping and cufflinks transcript assembly, a total of 222,031 unique transcripts were assembled from the high-throughput RNA-Seq data. To distinguish lncRNA candidates, five sequential stringent filters to the transcripts were employed (Supplementary Figure 2). First, a total of 209,311 transcripts with two or more exons were selected, and 12,720 transcripts were removed. LncRNAs are longer than 200 nucleotides in length, so 1,840 transcripts that were shorter than 200 bp were removed, and 207,471 transcripts were recovered in the second step. The transcripts which were overlapped with the annotated exons in the Genecode database were removed by using the Cuffcompare software, and 23,106 transcripts were obtained. Next, the expression level of every transcript was quantified by the software Cuffquant, and a total of 17,064 transcripts with FPKM ≥ 0.5 were obtained. These transcripts were further filtered using CNCI, CPC, Pfam-scan and phyloCSF to predict the coding potential. Eventually, this pipeline provided 10,315 lncRNAs (Supplementary Figure 2A).

According to their genome locations, these lncRNAs were further classified into three types: long intergenic ncRNAs (lincRNAs), intronic ncRNAs (incRNAs), and natural antisense transcripts (NATs) (Supplementary Figure 2B). We found that in cotton, the majority of lncRNAs (9,356, 90.7%) were lincRNAs, and only a small portion (959, 9.3%) of the lncRNAs were lncNATs. Furthermore, no intronic lncRNAs were uncovered in our database. These observations are consistent with previous studies (Wang et al., 2015a). The previous studies in zebrafish, *Arabidopsis*, maize, Populus and tomato have shown that lncRNAs harbor fewer exons and are shorter than protein-coding genes (Pauli et al., 2012; Li et al., 2014a; Shuai et al., 2014; Wang et al., 2014a; Zhu et al., 2015). To further identify whether cotton lncRNAs share these features, the density of exon number, length and the open reading frame (ORF) length of cotton lncRNAs were analyzed compared with the cotton mRNA (Supplementary Figures 2C–E). We found that the majority of the lncRNAs only contained one to two exons. In contrast, the number of exons for the protein-coding genes ranged from one to twenty (Supplementary Figure 2C). There is no significant difference between the length of lncRNAs and mRNAs (Supplementary Figure 2D). Most of the ORF lengths of lncRNAs were < 250 nucleotides, while the majority of the ORF lengths of mRNA were >250 nucleotides (Supplementary Figure 2E). Taken together, compared with the protein-coding genes, the majority of the lncRNAs in cotton contain fewer exons, and are relatively shorter.

### Genomic Landscape of Cotton Long Non-coding RNAs

**Figure 3:**
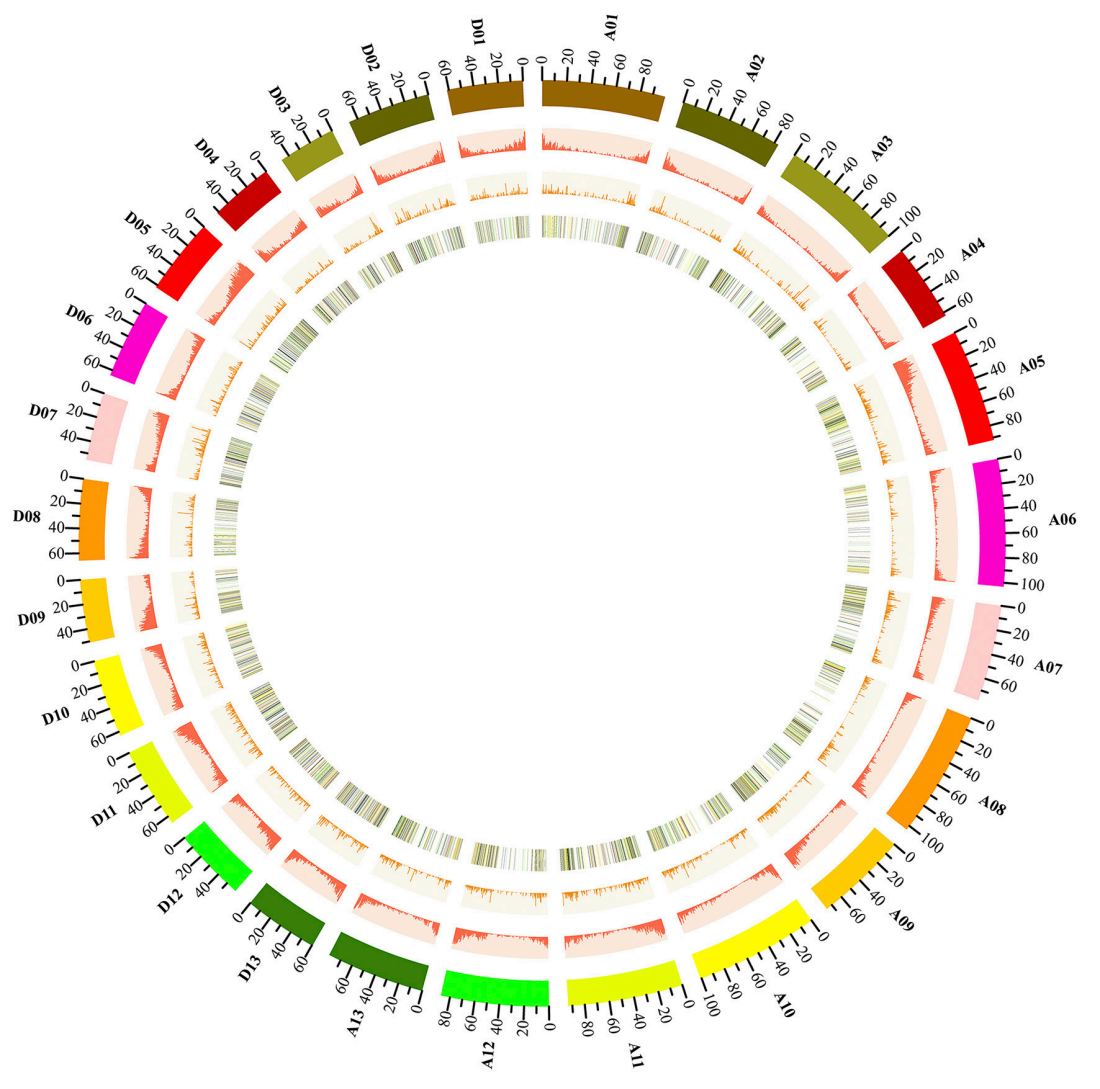
Genome-wide distribution of cotton lncRNAs compared with that of protein-coding genes. Chromosomes A0-13 and D0-13 are shown with different colors and in a circular form as the outer thick track. The chromosome scale (Mb) is labeled on each chromosome. On the second track (outer to inner), the red columns show the abundance of protein-coding genes in physical bins of 500-kb for each chromosome. For the third track, the orange columns show the abundance of lncRNAs in 500-kb windows. On the inner track is the heat map showing the ratio of averaged fragments per kilobase of exon per million fragments mapped (FPKM) values (AZD/DMSO) of lncRNA. Each vertical line on the inner track reports the location of lncRNAs throughout the whole cotton genome; the black lines represent lncRNAs with a fold change of FPKM < 0.5, the green lines show the fold change of FPKM >0 and < 1; the yellow lines show the fold change of FPKM ≥1 and < 2; the red lines show the fold change of FPKM ≥2.

Next, we further analyzed the genomic landscape of cotton lncRNAs (Figure 3). A total of 10,315 lncRNAs were identified in the present study. 7,640 of these lncRNAs were mapped onto chromosomes of the recently released cotton reference genome (Yu et al., 2014). The remaining 2,670 lncRNAs were only mapped onto scaffolds. A Circos plot clearly showed that the cotton lncRNAs were widely transcribed from every chromosome. We found that lncRNAs and protein-coding genes shared similar density distributions, which are both lower in the pericentromeric heterochromatin regions than in the euchromatin. This result indicated that lncRNAs may share similar transcription patterns with the protein-coding genes, which is just the same as reported in the previous studies (Wang et al., 2015a). Furthermore, the expression levels of lncRNAs which mapped onto chromosomes were analyzed. LncRNAs with FPKM ≥0.5 were selected. A total of 528 lncRNAs were removed and 7,012 lncRNAs were obtained. Fold changes (FPKM_AZD_/FPKM_DMSO_) of these lncRNAs were calculated and are shown by the color of the lines on the inner track of Figure 3. The fold changes of 623 lncRNAs were ≥2 and are shown by red lines. The yellow lines represent 3,857 lncRNAs with fold change ≥1 and < 2. lncRNAs with fold change < 1 are depicted by green lines. The inner track provided a genomic landscape of lncRNA expression levels.

### Differentially Expressed lncRNA Identification, GO and KEGG Enrichment Analysis of These lncRNAs Target mRNAs

**Figure 4:**
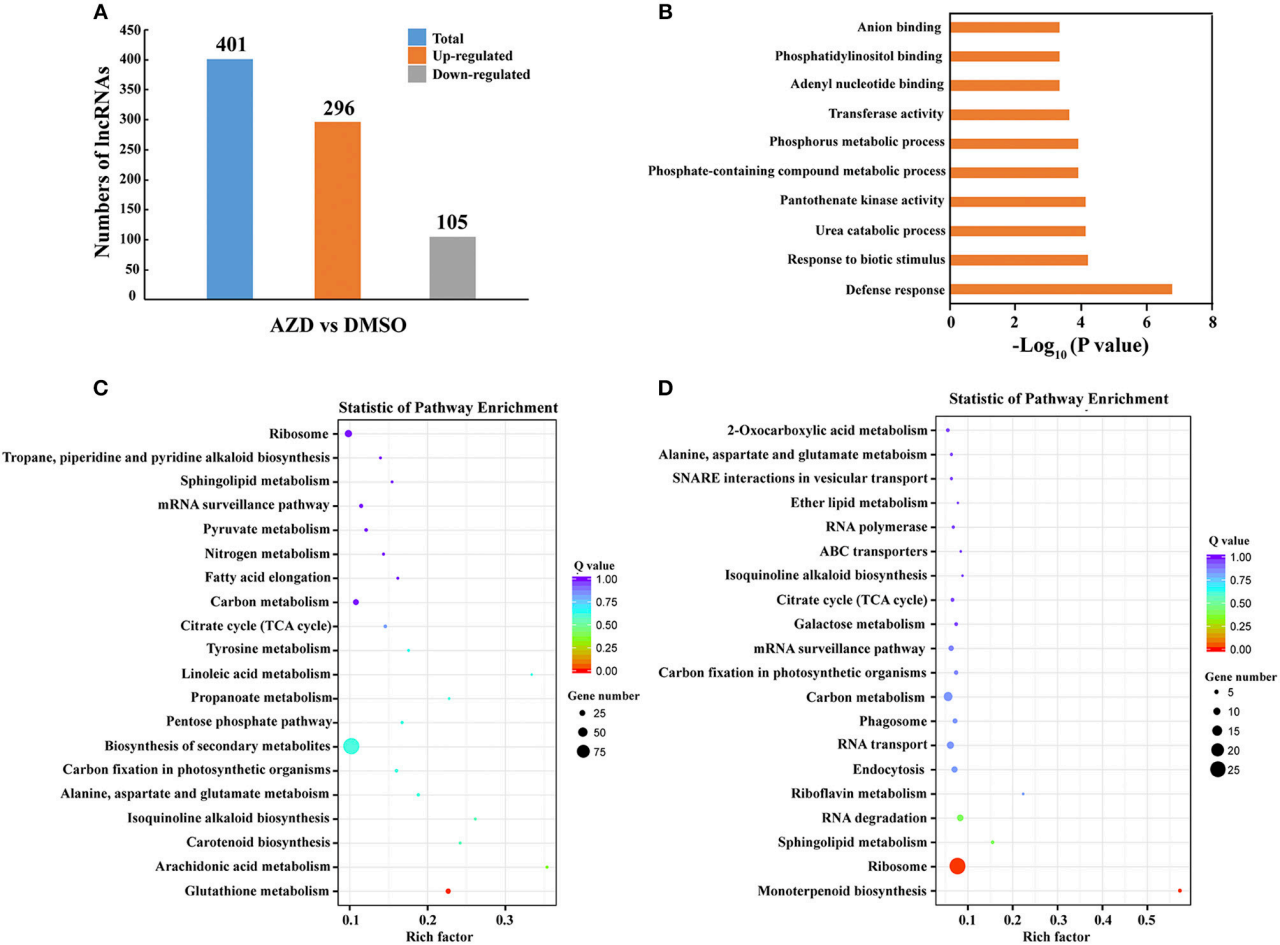
Differential expression of lncRNAs between DMSO and AZD treatment. **(A)** Compared with those in DMSO control, 296 lncRNAs were upregulated and 105 lncRNAs were downregulated in AZD treatment. **(B)** The top 10 enriched Gene ontology (GO) terms of differentially expressed lncRNAs. **(C)** Statistical KEGG enrichment of lncRNA target genes for upregulated differentially expressed lncRNAs using KOBAS software. **(D)** Statistical KEGG enrichment of lncRNA target genes for downregulated differently expressed lncRNAs using KOBAS software.

To identify the lncRNAs regulated by the TOR signaling pathway, the expression levels of lncRNAs were compared between DMSO control and AZD treatment in cotton seedlings. Following the procedure indicated in the methods, a total of 401 significantly differentially expressed lncRNAs were uncovered. Compared with the DMSO control, 296 of 401 lncRNAs were up-regulated in the cotton seedlings exposed to AZD, and the other 105 lncRNAs were downregulated (Figure 4A). To validate these differentially expressed lncRNAs, we randomly selected 10 of them, five were downregulated and five were upregulated. Their expression level changes indicated in RNA-seq data were validated by QRT–PCR experiments (Supplementary Figure 3). We also found that the expression patterns of these lncRNAs were consistent with that under TOR knockdown by VIGS.

Until now, the detailed mechanism of the interaction between mRNA and lncRNA is not clear. In previous studies, lncRNAs have been shown to preferentially locate next to genes with diverse functions (Dinger et al., 2008; Mercer et al., 2008; Guttman et al., 2009; Ponjavic et al., 2009; Cabili et al., 2011; Pauli et al., 2012; Wang et al., 2015a, 2018b). Thus, the protein-coding genes that are upstream or downstream 100 kb from the differentially expressed lncRNAs were selected to the Gene Ontology (GO) term analysis, and the top 10 enriched GO terms are shown in Figure 4B. The top two enriched GO terms were “defense response” and “response to biotic stimulus,” suggesting the potential function of the TOR signaling pathway in defense response through regulating cotton lncRNAs. The significantly differentially expressed lncRNAs potentially related to stress response are shown in Table 2, and a total of 21 lncRNAs were identified. We found that the lncRNAs that target the MLP-like protein 423 family, PYL and PP2C family may be regulated by TOR in cotton. KEGG (Kyoto Encyclopedia of Genes and Genomes) pathway analysis was also performed (Figures 4C,D). The top three enriched KEGG pathways of upregulated lncRNAs were “glutathione metabolism,” “arachidonic acid metabolism,” and “carotenoid biosynthesis.” The top three enriched KEGG pathways of down-regulated lncRNAs were “monoterpenoid biosynthesis,” “ribosome,” and “sphingolipid metabolism.”

**Table 2:** Differentially expressed lncRNA potentially involved in plant stress response.

**Transcript_id**	**Log_**2**_ (Fold change)**	**Q value**	**Target_gene_id**	**Target_description**
LNC_000377	−2.10225	5.08E-04	Gh_A02G0511	MLP-like protein 423
LNC_000377	−2.10225	5.08E-04	Gh_A02G0510	MLP-like protein 423
LNC_000377	−2.10225	5.08E-04	Gh_A02G0507	MLP-like protein 423
LNC_000377	−2.10225	5.08E-04	Gh_A02G0508	MLP-like protein 423
LNC_000377	−2.10225	5.08E-04	Gh_A02G0509	MLP-like protein 423
LNC_000378	−1.65483	4.20E-03	Gh_A02G0508	MLP-like protein 423
LNC_000378	−1.65483	4.20E-03	Gh_A02G0509	MLP-like protein 423
LNC_000378	−1.65483	4.20E-03	Gh_A02G0510	MLP-like protein 423
LNC_000378	−1.65483	4.20E-03	Gh_A02G0511	MLP-like protein 423
LNC_000512	−1.21876	1.31E-02	Gh_A02G0503	PYL11
LNC_000512	−1.21876	1.31E-02	Gh_A02G0504	MLP-like protein 423
LNC_000512	−1.21876	1.31E-02	Gh_A02G0505	MLP-like protein 423
LNC_000512	−1.21876	1.31E-02	Gh_A02G0498	MLP-like protein 423
LNC_000512	−1.21876	1.31E-02	Gh_A02G0500	MLP-like protein 423
LNC_000512	−1.21876	1.31E-02	Gh_A02G0508	MLP-like protein 423
LNC_000512	−1.21876	1.31E-02	Gh_A02G0502	MLP-like protein 423
LNC_000512	−1.21876	1.31E-02	Gh_A02G0499	MLP-like protein 423
LNC_000512	−1.21876	1.31E-02	Gh_A02G0495	PP2C family protein
LNC_000513	−1.05715	2.49E-03	Gh_A02G0499	MLP-like protein 423
LNC_000513	−1.05715	2.49E-03	Gh_A02G0495	PP2C family protein
LNC_000513	−1.05715	2.49E-03	Gh_A02G0502	MLP-like protein 423
LNC_000513	−1.05715	2.49E-03	Gh_A02G0508	MLP-like protein 423
LNC_000513	−1.05715	2.49E-03	Gh_A02G0503	PYL11
LNC_000513	−1.05715	2.49E-03	Gh_A02G0505	MLP-like protein 423
LNC_000513	−1.05715	2.49E-03	Gh_A02G0498	MLP-like protein 423
LNC_000513	−1.05715	2.49E-03	Gh_A02G0500	MLP-like protein 423
LNC_000513	−1.05715	2.49E-03	Gh_A02G0504	MLP-like protein 423
LNC_000513	−1.05715	2.49E-03	Gh_A02G0507	MLP-like protein 423
LNC_000513	−1.05715	2.49E-03	Gh_A02G0499	MLP-like protein 423
LNC_001145	−1.42464	1.83E-02	Gh_A05G1085	Homeobox-leucine zipper family protein
LNC_001145	−1.42464	1.83E-02	Gh_A05G1078	Methyl esterase 12
LNC_001977	−1.3256	5.08E-04	Gh_A07G1707	UDP-glucose 4-epimerase
LNC_002578	−1.87479	2.02E-02	Gh_A09G0802	Ankyrin repeat family protein
LNC_003270	−3.61046	2.45E-02	Gh_A11G1518	Plastidic GLC translocator
LNC_003666	1.51321	4.03E-02	Gh_A02G0509	MLP-like protein 423
LNC_004563	−1.24837	5.08E-04	Gh_D02G0574	MLP-like protein 423
LNC_004563	−1.24837	5.08E-04	Gh_D02G0569	MLP-like protein 423
LNC_004563	−1.24837	5.08E-04	Gh_D02G0570	MLP-like protein 423
LNC_004563	−1.24837	5.08E-04	Gh_D02G0571	MLP-like protein 423
LNC_004563	−1.24837	5.08E-04	Gh_D02G0567	Major allergen Pru ar 1
LNC_004563	−1.24837	5.08E-04	Gh_D02G0566	MLP-like protein 423
LNC_004695	−1.51079	5.08E-04	Gh_D02G0558	MLP-like protein 423
LNC_004695	−1.51079	5.08E-04	Gh_D02G0569	MLP-like protein 423
LNC_004695	−1.51079	5.08E-04	Gh_D02G0555	PP2C family protein
LNC_004695	−1.51079	5.08E-04	Gh_D02G0562	MLP-like protein 423
LNC_004695	−1.51079	5.08E-04	Gh_D02G0567	Major allergen Pru ar 1
LNC_004695	−1.51079	5.08E-04	Gh_D02G0560	MLP-like protein 423
LNC_004695	−1.51079	5.08E-04	Gh_D02G0566	MLP-like protein 423
LNC_004695	−1.51079	5.08E-04	Gh_D02G0559	Major allergen Pru ar 1
LNC_004696	−1.50013	5.08E-04	Gh_D02G0555	PP2C family protein
LNC_004696	−1.50013	5.08E-04	Gh_D02G0569	MLP-like protein 423
LNC_004696	−1.50013	5.08E-04	Gh_D02G0562	MLP-like protein 423
LNC_004696	−1.50013	5.08E-04	Gh_D02G0558	MLP-like protein 423
LNC_004696	−1.50013	5.08E-04	Gh_D02G0570	MLP-like protein 423
LNC_004696	−1.50013	5.08E-04	Gh_D02G0571	MLP-like protein 423
LNC_004696	−1.50013	5.08E-04	Gh_D02G0559	Major allergen Pru ar 1
LNC_004696	−1.50013	5.08E-04	Gh_D02G0560	MLP-like protein 423
LNC_004696	−1.50013	5.08E-04	Gh_D02G0567	Major allergen Pru ar 1
LNC_004696	−1.50013	5.08E-04	Gh_D02G0566	MLP-like protein 423
LNC_004697	−1.08682	2.36E-02	Gh_D02G0560	MLP-like protein 423
LNC_004697	−1.08682	2.36E-02	Gh_D02G0559	Major allergen Pru ar 1
LNC_004697	−1.08682	2.36E-02	Gh_D02G0555	PP2C family protein
LNC_004697	−1.08682	2.36E-02	Gh_D02G0569	MLP-like protein 423
LNC_004697	−1.08682	2.36E-02	Gh_D02G0571	MLP-like protein 423
LNC_004697	−1.08682	2.36E-02	Gh_D02G0570	MLP-like protein 423
LNC_004697	−1.08682	2.36E-02	Gh_D02G0558	MLP-like protein 423
LNC_004697	−1.08682	2.36E-02	Gh_D02G0562	MLP-like protein 423
LNC_004697	−1.08682	2.36E-02	Gh_D02G0566	MLP-like protein 423
LNC_004697	−1.08682	2.36E-02	Gh_D02G0567	Major allergen Pru ar 1
LNC_004698	−1.82071	5.08E-04	Gh_D02G0574	MLP-like protein 423
LNC_004698	−1.82071	5.08E-04	Gh_D02G0569	MLP-like protein 423
LNC_004698	−1.82071	5.08E-04	Gh_D02G0570	MLP-like protein 423
LNC_004698	−1.82071	5.08E-04	Gh_D02G0567	Major allergen Pru ar 1
LNC_004698	−1.82071	5.08E-04	Gh_D02G0558	MLP-like protein 423
LNC_004698	−1.82071	5.08E-04	Gh_D02G0562	MLP-like protein 423
LNC_004698	−1.82071	5.08E-04	Gh_D02G0566	MLP-like protein 423
LNC_004698	−1.82071	5.08E-04	Gh_D02G0571	MLP-like protein 423
LNC_004698	−1.82071	5.08E-04	Gh_D02G0559	Major allergen Pru ar 1
LNC_004698	−1.82071	5.08E-04	Gh_D02G0560	MLP-like protein 423
LNC_005100	−1.63418	4.01E-02	Gh_D04G1789	Anthranilate phosphoribosyltransferase
LNC_005163	−3.74603	1.64E-02	Gh_D04G1399	Pathogenesis-related protein
LNC_005763	−2.47174	5.08E-04	Gh_D07G0796
LNC_005731	−1.08659	9.52E-04	Gh_D07G0193	PYR1-like 2
LNC_006174	−1.80347	9.75E-03	Gh_D08G0260	MLO family protein
LNC_007115	1.87584	2.79E-02	Gh_D04G1399	Pathogenesis-related protein
LNC_009095	1.63788	1.58E-02	Gh_D02G0555	PP2C family protein
LNC_009095	1.63788	1.58E-02	Gh_A02G0495	PP2C family protein

### Differentially Expressed lncRNAs Involved in Plant Growth

As the phenotypes indicated in the TOR-inhibited and TOR-silenced cotton seedlings (Figures 1D, 2A), cotton seedling growths were significantly retarded. Plant cell growth is tightly linked to ribosome biogenesis and photosynthesis (Dong et al., 2015). The ribosome is a key component of cell growth control and protein synthesis. The “Ribosome” and “Ribosome biogenesis in eukaryotes” pathways were identified in the enriched downregulated KEGG pathways. LncRNAs involved in this process were listed in Supplementary Table 4A. A total of 37 downregulated lncRNAs were enriched in this pathway. Ribosomal protein (RP) is one of the important components of the ribosome (Ben-Shem et al., 2011). Twenty-nine of these downregulated lncRNAs targeted genes encoded ribosomal proteins, indicating the important role of TOR in regulating the cotton ribosome biogenesis. Transposons associate with the origins and transcribed regions of lncRNAs and eventually drive lncRNA expression (Ponting et al., 2009; Kapusta et al., 2013; Xu et al., 2015; Wang et al., 2016a,b; Sherafatian and Mowla, 2017). We analyzed the potential transposons that targeted these ribosome-related lncRNAs (Supplementary Table 4B). Transposons were identified in the sequences of LNC_000574, LNC_000642, LNC_004307, LNC_006480, LNC_006613 and LNC_007286 by WUBlastX. Most of the identified transposons were long terminal repeat retrotransposons (LTR), and three of them were LTR/copia transposons. These results indicated that transposon-related insertions may play a role in regulating lncRNA expression. One of the important functional patterns seen for lncRNAs is the interplay between miRNAs and lncRNAs, and lncRNAs could be targeted by miRNAs (Yoon et al., 2014). To identify whether lncRNAs are bona fide targets for miRNAs, we analyzed the selected lncRNAs associated with ribosomes using psRNATarget (Supplementary Table 4C) (Dai and Zhao, 2011). Twenty-four of the ribosome-related lncRNAs were targeted by miRNAs, and a total of 52 miRNAs were identified. In addition, one miRNA had multiple targets. For example, in the case of ghr-miR2949a-3p, it not only targeted LNC_000642 but also LNC_005100 and LNC_009692.

As one of the most important anabolic processes, photosynthesis also plays a vital role in plant cell growth. The TOR-inhibited and silenced cotton seedlings showed a significant delay in the transition from heterotrophic to photoautotrophic growth and exhibited pale yellow-green and shrinking leaves, which indicated the affected photosynthesis in cotton. The enriched KEGG pathways “carbon fixation in photosynthetic organism” and “photosynthesis” were also detected. Fifteen genes targeted by these down-regulated lncRNAs were found in these pathways and are displayed in Supplementary Table 5A. Genes encoded, including phosphoglycerate kinase (PGK1 and PGK3), alanine aminotransferase 2 (ALAAT1), aspartate aminotransferase 3 (ASP3), photosystem II reaction center protein K precursor, cytochrome c and photosynthetic electron transfer A, were found. Additionally, transposons and miRNAs were analyzed in the sequences of these lncRNAs. As displayed in Supplementary Table 5B, transposons were only identified in the sequences of LNC_004722. Furthermore, 26 miRNAs were found to target these photosynthesis-related lncRNAs, and eight of these lncRNAs were targeted by the miRNAs (Supplementary Table 5C). The above results further supported the phenotypes indicated in the TOR-inhibited cotton seedlings and the conserved functions of TOR as reported in other species (Martin et al., 2004; Ren et al., 2011; Dong et al., 2015).

### Expression Pattern Analysis of *GhTOR* Genes Under Different Stresses

**Figure 5:**
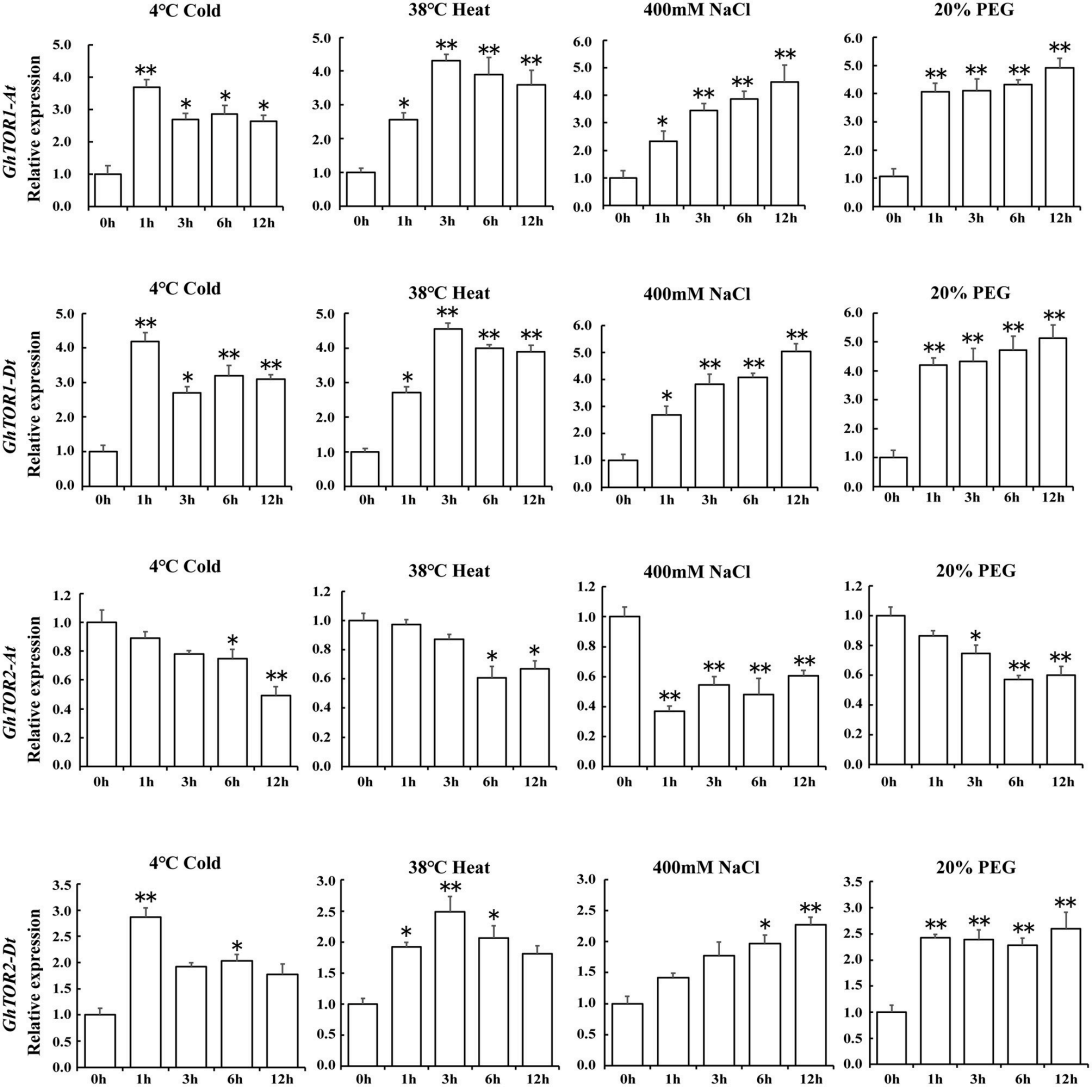
Gene expression profiles of *TOR* genes under different stresses. Error bar represents the standard deviation of three independent experiments. Asterisks (^**^p < 0.01, ^*^p < 0.05) indicate significant differences from the control.

As indicated in the GO terms analysis of differentially expressed lncRNAs, defense response-related GO terms were enriched. Environmental stresses significantly influenced plant growth and finally caused plant biomass losses, which were similar to the phenotypes indicated in the *TOR*-silenced cotton seedlings, including wilting and decreased fresh weights (Figure 2). Therefore, the expression patterns of *GhTOR* genes under abiotic stress conditions, i.e., cold, heat, salt, and drought were assessed, and the corresponding results were shown in Supplementary Figure 1C and Figure 5. The expression levels of *GhTOR* genes in Figure 5 were similar to the expression patterns shown in Supplementary Figure 1C. The expression levels of *GhTOR* genes were induced by diverse stresses, indicating that TOR responded to multiple stresses. When responding to different stresses, the expression levels of *GhTOR1-At, GhTOR1-Dt*, and *GhTOR2-Dt* increased; however, the transcript levels of *GhTOR2-At* were down-regulated. In response to cold, the expression levels of *GhTOR1-At, GhTOR1-Dt*, and *GhTOR2-Dt* reached a peak at 1 h, then maintained a relatively high and stable level. Meanwhile, in response to heat they reached a peak at 3 h. The expression levels of these three genes showed a gradual growth trend when responding to NaCl and PEG treatments. These results indicated that *TOR* genes may be involved and play potentially different roles in cotton stress response.

### Differentially Expressed lncRNAs Participated in Plant Stress Response

Expression pattern analysis of *TOR* genes indicated that *TOR* genes responded to different stress treatments (Figure 5 and Supplementary Figure 1). In our previous study, the differentially expressed protein coding genes of AZD-treated cotton seedlings were analyzed and identified (Song et al., 2017). The Pearson correlation coefficient was employed to explore the expression relationship between these lncRNAs and neighboring protein-coding genes, six (r_p_ > 0.95) of these lncRNAs were selected, including LNC_00512, LNC_00513, LNC_004695, LNC_004696, LNC_004697, and LNC_004698. LNC_00513 belonged to lincRNA, and the other five lncRNAs belonged to antisense lncRNAs. We found that the expression levels of the lncRNA-targeted genes Gh_A02G0500 and Gh_D02G0560, which both encoded MLP-like protein 423, were significantly upregulated in response to TOR inhibition, with the fold change of 14.8 and 5.1, respectively. As indicated in Table 2, Gh_A02G0500 was targeted by LNC_00512 and LNC_00513, and Gh_D02G0560 was targeted by LNC_004695, LNC_004696, LNC_004697, and LNC_004698. The expression levels of these above lncRNAs were downregulated under TOR inhibition with the fold changes varying from 0.2 to 0.4, indicating that TOR may be a positive regulator of these lncRNAs. Under TOR inhibition, these lncRNAs were downregulated, whereas their targets Gh_A02G0500 and Gh_D02G0560 were upregulated, suggesting the possibility that these lncRNAs may act as a negative regulator in the transcript process of these two genes. In the sequences of LNC_004698, six potential transposons were identified (Supplementary Table 6A) and all of them belonged to LINE/L1, indicating the potential role of transposons in regulating LNC_004698 expression. In the analysis of these stress related lncRNAs, we found 30 miRNAs targeted these lncRNAs, and 15 of these lncRNAs were targeted by miRNAs (Supplementary Table 6B). LNC_005731 and LNC_006174 were the target of ghr-miR399, which is engaged in salt tolerance of cotton (Deng et al., 2018).

**Figure 6:**
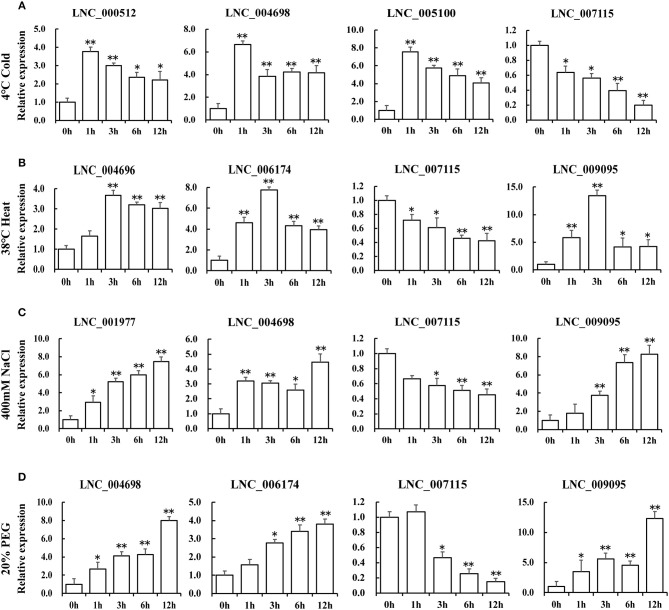
Expression levels of differentially expressed lncRNAs under cold **(A)**, high temperature **(B)**, NaCl **(C)**, and PEG **(D)** treatments. Error bars represent the standard deviation of three independent experiments. Asterisks (^**^p < 0.01, ^*^p < 0.05) indicate significant differences from the control.

To characterize the putative function of these lncRNA candidates in response to different stresses in Table 2, the expression levels of lncRNAs under the same stress treatments have been identified by QRT-PCR (Figure 6). We selected four lncRNAs that showed similar expression patterns with *TOR* genes for every stress treatment (Figure 6). In response to cold treatment, LNC_000512, LNC_004698, LNC_005100 showed a quick response and peaked at 1 h. In response to heat stress, LNC_004696, LNC_006174 and LNC_009095 reached the maximum at 3 h. We found that LNC_001977, LNC_004698, LNC_006174, and LNC_009095 displayed similar expression patterns when exposed to NaCl and PEG treatments, indicating that they may play a similar role in salinity and drought response. When responding to diverse stresses, all the transcript levels of LNC_007115 were downregulated, indicating that this lncRNA may be a negative regulator of stress response and may be regulated by *GhTOR2-At*, which showed a similar expression pattern. Gene expression profiles of these putative stress-related lncRNAs suggested that they may function as regulators downstream of TOR.

## Discussion

The TOR (target of rapamycin) signaling pathway functions as a key player in integrating stress-related cues with growth (Rexin et al., 2015; Shi et al., 2018). However, little is known about the molecular mechanism of how the TOR signaling pathway regulates plant stress in the tetraploid cotton. In the present study, we characterized the functions of cotton TOR and performed the comprehensive analysis of *TOR* genes in cotton and the lncRNAs regulated by TOR signaling, with the aim of obtaining a better understanding of their functional roles in future studies.

In our previous research, we revealed that the TOR signaling pathway existed in tetraploid cotton and also analyzed the cotton transcriptome under TOR inhibition (Song et al., 2017). In this study, functional analysis was further performed to elucidate the role of TOR protein in cotton. Due to the difficulties in genetic transformation in cotton, VIGS (Virus-induced gene silencing) technology is used to identify cotton gene functions as a result of its straightforward operation, quick returns and free transformation (Yang et al., 2015; Cheng et al., 2016). Similar to the phenotypes applied by TOR inhibitor AZD treatment (Figure 1D), knockdown of *GhTOR* genes caused pale yellow-green and shrinking leaves (Figure 2), which was consistent with the previous report that TOR played a role in regulating cotyledon greening and photosynthesis (Dong et al., 2015; Li et al., 2015b). The KEGG analysis of lncRNAs in Figure 4 further confirmed the conserved role of TOR.

Emerging evidence supports the view that lncRNAs play important roles in many fundamental biological processes (Liu et al., 2015). In plants, systematic searches of lncRNAs have been conducted in *Arabidopsis*, wheat, rice, *Zea mays*, tomato and cotton fibers (Li et al., 2007, 2014a; Ben Amor et al., 2009; Xin et al., 2011; Liu et al., 2012; Wang et al., 2014a, 2015a; Zhu et al., 2014; Hu et al., 2015). However, much work still remains to be done with cotton. In the present study, through utilizing the similar strict criteria pipelines to identify lncRNAs used in previous studies in other plants (Sun et al., 2013; Zhu et al., 2014), a total of 10,315 lncRNAs were identified in cotton, of which 9,356 were lincRNAs and 959 were lncNATs (Supplementary Figure 2B). Compared with the previous lncRNA studies in cotton, the identified lncRNA numbers in our study were far less than those of Wang et al. (Wang et al., 2015a) but were similar to those in Lu et al. (2016). This may be due to the more rigorous filtration criteria we used to identify lncRNAs. Although some cotton lncRNAs might be excluded due to the sequencing limitations and strict bioinformatics criteria, a relatively reliable list of cotton lncRNAs is provided, which could be useful for other researchers. None of the intronic lncRNAs were identified in our database, which is different from the previous studies in cotton. We speculate that intronic lncRNAs may be dispensable in the early stage of cotton seedling growth.

Great progress has been made in dissecting the function of the TOR signaling pathway in regulating lncRNAs in humans (Ge et al., 2014; Li et al., 2014b; Wang et al., 2015b; Yacqub-Usman et al., 2015; Wu and Luo, 2016). Yet, functional characterization of the TOR signaling pathway in regulating plant lncRNAs is still largely uncovered. In this study, we identified a total of 401 differentially expressed lncRNAs in the TOR-inactivated cotton seedlings, which might be regulated by the TOR signaling pathway. These differentially expressed lncRNAs represent functional candidates in future experimental studies. Compared with animals, photosynthesis is one of the unique features of plants. VIGS of *GhTOR* genes in cotton resulted in a compromised photosynthetic ability (yellow-green and shrinking leaves). Previous expression profiling and functional analysis revealed that TOR is a key player in regulating photosynthesis (Dong et al., 2015). In our study, the enriched KEGG pathway “carbon fixation in photosynthetic organism” and “photosynthesis” further confirmed the role of TOR signaling pathway in plant photosynthesis. TOR signaling pathway also is a highly conserved regulator of eukaryotic stress resistance. Studies in *Arabidopsis* revealed that TOR-dependent downregulation of S6K activity was integral to adaptive osmotic stress responses (Mahfouz et al., 2006; Deprost et al., 2007). Furthermore, TOR-dependent regulation of autophagy occurred either downstream or independently of ROS-related signaling inputs, which were highly associated with stresses (Liu and Bassham, 2010). An ancient regulatory module, Snf1 (sucrose-non-fermenting 1), communicated with cellular energy levels and associated stresses to downstream targets, including TOR (Hardie et al., 2012). In our study, GO enrichment analysis revealed that neighbor protein-coding genes of differentially expressed lncRNAs were significantly enriched in defense response, indicating the potential role of these lncRNAs in plant stress responses. In Table 2, a mass of MLP (Major Latex Protein) family proteins are identified. A number of studies have suggested that MLP protein is related to biotic and abiotic stresses (Schenk et al., 2000; Qu et al., 2005; Siemens et al., 2006; Chen and Dai, 2010; Malter and Wolf, 2011; Wang et al., 2011; Yang et al., 2015). Further studies may be needed to dissect the molecular basis of the relationship between these lncRNAs and TOR in stress response. Wang et al. revealed that the TOR kinase and ABA receptor balanced plant growth and stress response (Wang et al., 2018a). Consistently, two ABA receptors, PYL2 and PYL11, were also targeted by differentially expressed lncRNAs in our results. Transcription analysis of differentially expressed lncRNAs under multiple stresses further confirmed the involvement of these lncRNAs in plant stress response (Figure 6). Taken together with the expression patterns of *GhTOR* genes under different stresses (Figure 5 and Supplementary Figure 1), our results indicated that TOR may directly regulate these potential stress-related lncRNAs and play a vital role in plant stress response. In addition, it is well-known that transposons associate with lncRNA origins and lncRNAs' transcribed regions, eventually driving expression of lncRNAs, and microRNA can also target lncRNAs (Ponting et al., 2009; Kapusta et al., 2013; Xu et al., 2015; Wang et al., 2016a,b; Sherafatian and Mowla, 2017). To confirm the lncRNAs' functions selected in the present study, transposons and miRNAs related with these selected lncRNAs were analyzed (Supplementary Tables 4–6).

In summary, this is the first study to characterize cotton lncRNAs potentially regulated by the TOR signaling pathway. As a key upstream protein kinase, TOR initiates many downstream signaling cascades in all examined eukaryotic species. TOR protein should have many downstream signaling proteins and transcriptional factors, which may regulate the expression of lncRNAs in plants. Functional characterization of lncRNAs is still in its infancy. Further studies should be conducted to elucidate the biological functions of these candidate lncRNAs and how they are regulated by the TOR signaling pathway. Our study provides new information that underpins the functional characterization of lncRNAs potentially regulated by the TOR signaling pathway in cotton. These data provide a useful resource of lncRNAs and a foundation for functional research of TOR in cotton stress responses.

## Author Contributions

FL, MR, and YS designed the experiments; YS, LL, ZhY, GZ, XZ, FZ, XG, CZ, LZ, LW, HY and ZuY performed the experiments; YS, LL, and ZhY analyzed the data; FL, MR, and YS wrote the manuscript.

### Conflict of Interest Statement

The authors declare that the research was conducted in the absence of any commercial or financial relationships that could be construed as a potential conflict of interest.

## Supplementary Material

Supplementary Figure 1 Expression pattern analysis of *GhTOR* genes in different tissues **(A)** during seed germination **(B)** and under different stresses **(C)**. The expression levels of *GhTOR* genes were identified by utilizing the RNA-Seq expression profiles of *G.hirsutum* TM-1 (Zhang et al., 2015).

Supplementary Figure 2 Genome-wide identification and characterization of long non-coding RNAs (lncRNAs) in *Gossypium hirsutum*. **(A)** The detailed schematic diagram of the pipeline for lncRNA identification in *G. hirsutum*. **(B)** Classification of cotton lncRNAs according to its genomic position. The proportion of the three kinds of lncRNAs was calculated. The distribution of exon numbers **(C)**, lengths **(D)**, and ORF lengths **(E)** of lncRNAs in comparison with protein-coding transcripts of cotton.

Supplementary Figure 3 QRT–qPCR verification of RNA-Seq data of 10 randomly selected lncRNAs. Five downregulated and five upregulated lncRNAs according to the RNA-Seq data were quantified. *GhHistone3* expression values were used as the internal reference. Error bars indicate ±SD of three biological replicates, each measured in triplicate.

Supplementary Table 1 The list of lncRNAs detected in cotton seedlings.

Supplementary Table 2 The list of differentially expressed lncRNAs.

Supplementary Table 3 Primers used in this study.

Supplementary Table 4 **(A)** Differentially expressed lncRNAs involved in ribosome. **(B)** The predicted transposons targeted ribosome related lncRNAs in the transcriptome data. **(C)** Predicted miRNA targets.

Supplementary Table 5 **(A)** Differentially expressed lncRNAs involved in photosynthesis. **(B)** The predicted transposons targeted photosynthesis related lncRNAs in the transcriptome data. **(C)** Predicted miRNA targets.

Supplementary Table 6 **(A)** The predicted transposons targeted stress related lncRNAs in the transcriptome data. **(B)** Predicted miRNA targets.
