# Supplementary figures and images for: Target of Rapamycin (TOR) Regulates the Expression of lncRNAs in Response to Abiotic Stresses in Cotton
Source: Front Genet. 2019 Jan 8;9:690. doi: 10.3389/fgene.2018.00690 (PMC6332313; doi:10.3389/fgene.2018.00690)

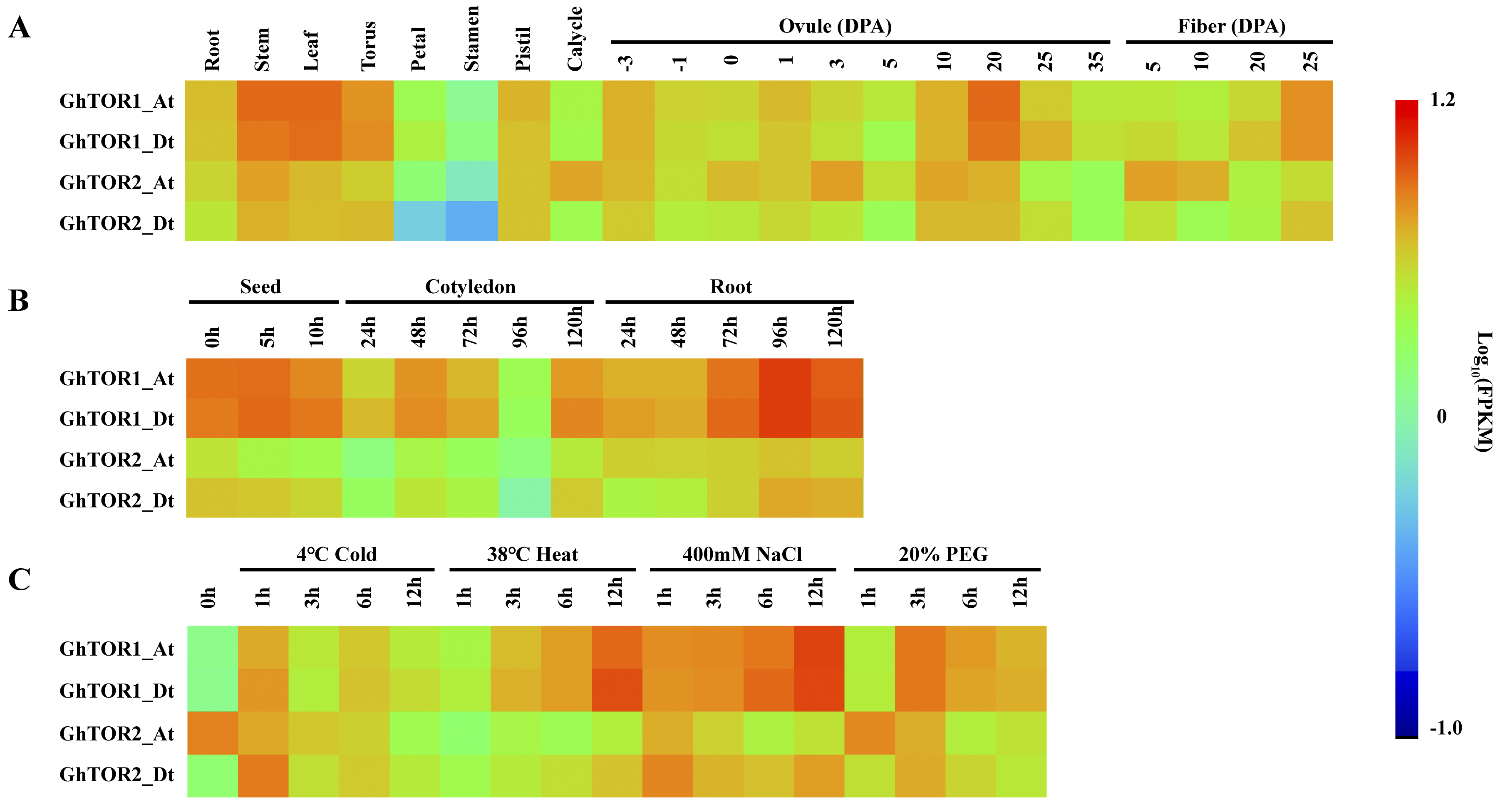

Supplement: Supplementary Figure 1 — Expression pattern analysis of GhTOR genes in different tissues (A) during seed germination (B) and under different stresses (C). The expression levels of GhTOR genes were identified by utilizing the RNA-Seq expression profiles of G.hirsutum TM-1 (Zhang et al., 2015). [file Image_1.TIF]

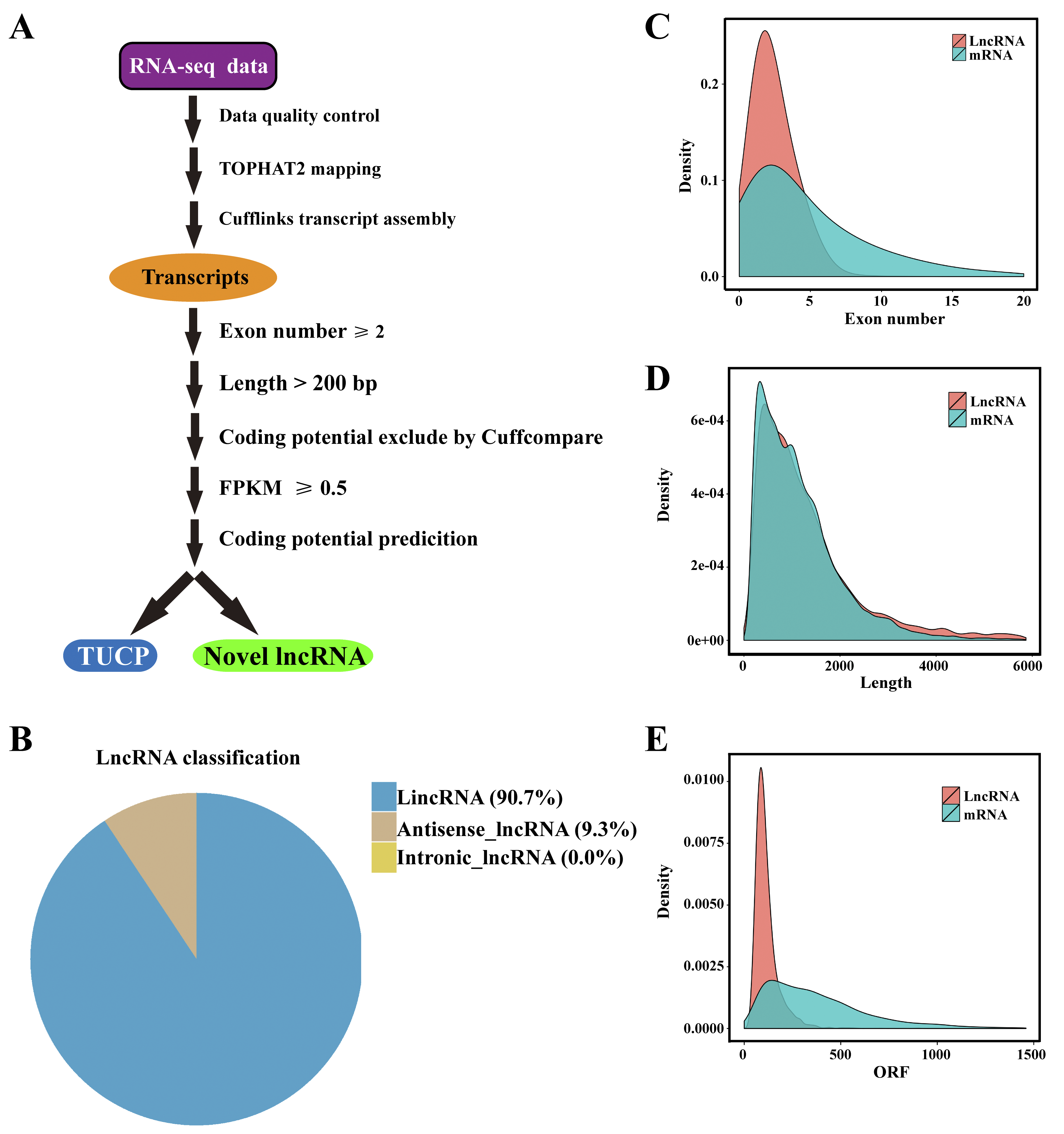

Supplement: Supplementary Figure 2 — Genome-wide identification and characterization of long non-coding RNAs (lncRNAs) in Gossypium hirsutum. (A) The detailed schematic diagram of the pipeline for lncRNA identification in G. hirsutum. (B) Classification of cotton lncRNAs according to its genomic position. The proportion of the three kinds of lncRNAs was calculated. The distribution of exon numbers (C), lengths (D), and ORF lengths (E) of lncRNAs in comparison with protein-coding transcripts of cotton. [file Image_2.TIF]

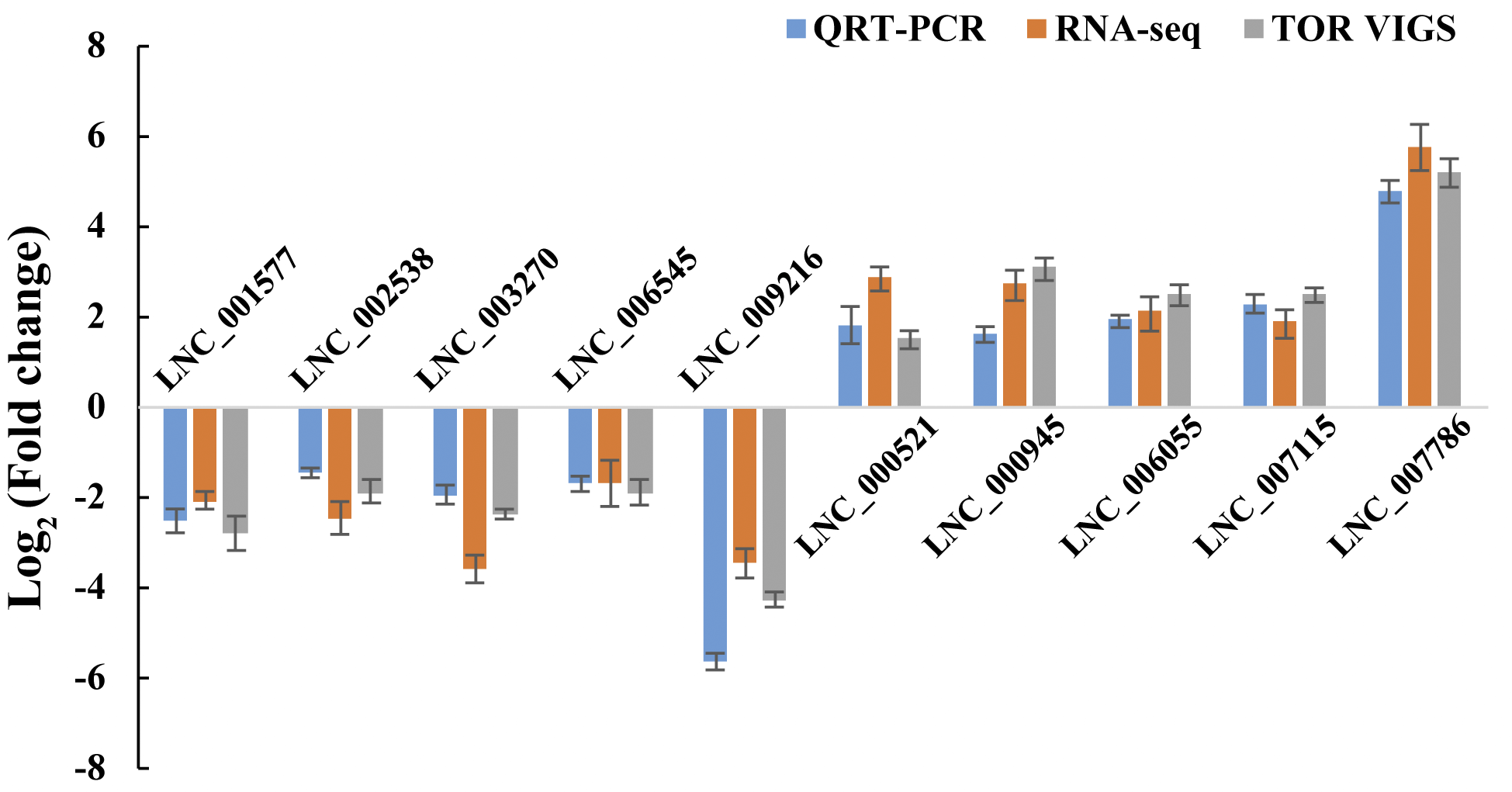

Supplement: Supplementary Figure 3 — QRT–qPCR verification of RNA-Seq data of 10 randomly selected lncRNAs. Five downregulated and five upregulated lncRNAs according to the RNA-Seq data were quantified. GhHistone3 expression values were used as the internal reference. Error bars indicate ±SD of three biological replicates, each measured in triplicate. [file Image_3.TIF]
